# Gut microbiota in acute leukemia: Current evidence and future directions

**DOI:** 10.3389/fmicb.2022.1045497

**Published:** 2022-12-01

**Authors:** Yao Zhou, Chendan Zhou, Aijun Zhang

**Affiliations:** Department of Pediatrics, Qilu Hospital of Shandong University, Jinan, China

**Keywords:** gut microbiota, acute leukemia, chemotherapy, immunotherapy, hematopoietic stem cell transplantation, probiotics

## Abstract

Gut microbiota includes a large number of microorganisms inhabiting the human gastrointestinal tract, which show a wide range of physiological functions, including digestion, metabolism, immunity, neural development, etc., and are considered to play an increasingly important role in health and disease. A large number of studies have shown that gut microbiota are closely associated with the onset and development of several diseases. In particular, the interaction between gut microbiota and cancer has recently attracted scholars’ attention. Acute leukemia (AL) is a common hematologic malignancy, especially in children. Microbiota can affect hematopoietic function, and the effects of chemotherapy and immunotherapy on AL are noteworthy. The composition and diversity of gut microbiota are important factors that influence and predict the complications and prognosis of AL after chemotherapy or hematopoietic stem cell transplantation. Probiotics, prebiotics, fecal microbiota transplantation, and dietary regulation may reduce side effects of leukemia therapy, improve response to treatment, and improve prognosis. This review concentrated on the role of the gut microbiota in the onset and development of AL, the response and side effects of chemotherapy drugs, infection during treatment, and therapeutic efficacy. According to the characteristics of gut microbes, the applications and prospects of microbial preparations were discussed.

## Introduction

A large number of microorganisms, including bacteria, fungi, viruses, archaea, etc., are colonized in the human body surface and digestive tract, and the related genomes of these microorganisms are referred as microbiome (Fettweis et al., 2019). Among them, gut microbiota has the largest microbial community, including billions of types of microbes. The composition of gastrointestinal microbiota is influenced by heredity, environment, lifestyle, and other factors (Rothschild et al., 2018). Gut microbiota plays several roles in the host, such as digestion and absorption of food, synthesis of vitamins, short-chain fatty acids, essential amino acids and secondary metabolites, development and maturation of the immune system, involvement in the regulation of immune response and pathogen resistance, and even influencing our emotional and cognitive behaviors (de Vos et al., 2022).

Gut microbiota plays a fundamental role in maintaining homeostasis and intestinal integrity and is considered to play an increasingly important role in health and disease (Sekirov et al., 2010). In recent years, the gut microbiota has been reported to be closely correlated with various diseases, including intestinal diseases (e.g., inflammatory bowel disease), chronic metabolic diseases (e.g., diabetes), autoimmune diseases (e.g., rheumatoid arthritis), central nervous system diseases (e.g., Parkinson’s disease), respiratory diseases (e.g., asthma), carcinoma (e.g., hepatocellular carcinoma), etc. (Yu and Schwabe, 2017; Maeda and Takeda, 2019; Barcik et al., 2020; Gurung et al., 2020; Lavelle and Sokol, 2020; Kaur et al., 2021). Moreover, intestinal microbiota homeostasis and imbalance is an important factor affecting drug efficacy and side effects (Zhang et al., 2018). A growing body of evidence demonstrated that gut microbiota plays a role in the initiation, progression, and treatment of cancer. For instance, studies have shown that human microbiota is closely associated with the incidence of esophageal cancer, gastric cancer, hepatobiliary cancer, pancreatic cancer, colorectal cancer, lung cancer and other tapes of cancer (Vogtmann and Goedert, 2016).The present review, aimed to summarize the alterations of the gut microbiota in patients with acute leukemia (AL) and its effects on disease pathogenesis, progression, and treatment, and to discuss several approaches to improve leukemia outcomes by targeting the gut microbiota.

## Possible relationships between gut microbiota and the leukemia initiation

The human gut microbiota is a complex microbial system. Previous research in the Human Microbiome Project has shown that more than 2,000 species have been isolated from the human gut microbiome (Fettweis et al., 2019), while only few of them can be found in all humans (Qin et al., 2010). The colony is composed of various types of microbiotas, whereas *Bacteroides* and *Actinobacteria* account for about 90% of the gut microbiota (Qin et al., 2010; Mandal et al., 2015). It was reported that the composition of gut microbiota in the population has certain similarities, and the onset and development of diseases many affect the composition of the gut microbiota. Studies demonstrated that pre-treatment with composition of gut microbiota can differentiate healthy individuals from cancer patients. Several sterile animal studies have shown that the microbiota promotes tumors in various organs, including skin, colon, liver, breast, lung, ect. (Sacksteder, 1976; Uronis et al., 2009; Kang et al., 2021; Matson et al., 2021). The analysis of metagenomic samples from different cancer patients showed that microbiota may be used as diagnostic bacterial markers in diverse diseases, such as *Bacteroides fragilis* and *Fusobacterium nucleatum* in colorectal cancer, and the *kkermansia muciniphila*, *Rikenellaceae* and *Bacteroides* et al. in non-small cell lung cancer (Cheng et al., 2020; Vernocchi et al., 2020).

Several studies have shown that microbial dysbiosis was associated with the initiation of various tapes of cancer. To explore the relationship between gut microbiota and AL, it was attempted to summarize some of the studies on microbiota in leukemia (Table 1). Several clinical studies have demonstrated a structural imbalance in the gut microbiota of acute lymphoblastic leukemia (ALL) patients before treatment compared with healthy controls. Shannon index, which summarizes taxonomic richness (i.e., the number of species represented) and evenness (i.e., the degree to which species represented are similar in frequency), is one of the most common measures of alpha diversity (i.e., within-sample diversity) reported in the gut microbiota literature and is considered as a marker of the health of gut microbiota. This conclusion was confirmed by performing a meta-analysis of the Shannon index results from six studies (Supplementary Figures 1, 2). These findings verified the interaction between gut microbiota and AL initiation and progression. However, the results regarding the alterations of specific microbiota genus were inconsistent. It may be related to the inconsistent sequencing depth of different studies and the small sample size of a single study, indicating the necessity of more standardized large-scale deep sequencing samples for analysis in the future.

**Table 1:** Microbiota studies in Acute Leukemia.

Study	Country	leukemia typing	Patients (Male)	HC sex (M/F)	Seq. Region	Sampling time point	Significance
Rajagopala et al. (2016)	America	ALL	28 (19)	23 (17)	V1–V3	Baseline, During chemotherapy	The gastrointestinal microbiota profiles of the patients and their healthy siblings were compared. There were many common groups between the two groups, but the microbiota diversity of the patients group was significantly lower than that of the control group
Bai et al. (2017)	China	ALL	30 (19)	33 (18)	V3–V4	Baseline	ALL induced structural changes of the gut microbiota, with the alpha diversity being significantly weakened by antibiotics, but not beta diversity.Bacteroidales and Enterococcaceae can be referred to as biomarkers for ALL
Chua et al. (2017)	Malaysia	ALL	73 (28)	61 (28)	V4	End of the whole chemotherapy	Demonstrated a relationship between microbiota and immune dysregulation in adult ALL survivors
Hakim et al. (2018)	America	ALL	199 (118)	NA	V1–V3	Baseline, During chemotherapy	The relative abundance of Proteobacteria before chemotherapy initiation predicts development of febrile neutropenia, and domination of the gut microbiota by Enterococcaceae or Streptococcaceae at any time during chemotherapy predicts infection in subsequent phases of chemotherapy
Nearing et al. (2019)	Canada	ALL	16 (11)	NA	V4–V5	Baseline, During chemotherapy, IC	A significant difference in alpha diversity and beta diversity was found in the samples of patients with infectious complications during the first 6 months of treatment. The machine learning model based on patient clinical data and bacterial species was able to predict the infection of patients
Chua et al. (2020)	Malaysia	ALL	7 (6)	7 (6)	V4	Baseline, During chemotherapy, End of the whole chemotherapy	Even after chemotherapy was stopped, the distribution of gut microbiota in children with ALL remained slightly different from that in healthy controls
De Pietri et al. (2020)	Denmark	ALL	51 (13)	19 (9)	V3–V4	Baseline, During chemotherapy	Bacterial alpha diversity was lower in patients compared to siblings. It decreased from Day 1 to Days 8–22 and increased on Day 29
Galloway-Peña et al. (2020)	America	AML	97 (48)	NA	V4	IC	That gut microbiome evaluation could assist with infectious risk stratification and that improved targeting of antibiotic administration during IC could decrease subsequent infectious complications in AML patients
Gao et al. (2020)	China	ALL	18 (9)	18 (10)	V3–V4	Baseline, During chemotherapy	Compared to healthy controls, ALL patient showed significant changes of GI microfloras
Liu et al. (2020)	China	ALL	58 (34)	23 (11)	V1–V9	Baseline	The composition of gut microbiota differed from healthy controls to pediatric ALL patients
Rattanathammethee et al. (2020)	Thailand	AML	10 (4)	NA	V3–V4	Baseline, During chemotherapy, IC	Adult AML patients with a first episode of febrile neutropenia after initial intensive chemotherapy demonstrated a significant decrease in gut microbiota diversity and the level of diversity remained constant despite recovery of bone marrow
Bhuta et al. (2021)	America	ALL	9 (NA)	10 (NA)	V4	End of the whole chemotherapy	Bacterial strains differed between ALL patients and healthy siblings, and the gut microbiome composition remained different between childhood ALL survivors and healthy sibling controls
Rashidi et al. (2021)	America	ALL (4) and AML (16)	20 (9)	NA	V4	During chemotherapy	That Akkermansia expansion in the gut was associated with an increased risk for neutropenic fever
Shen et al. (2021)	China	ALL (11) and AML (9)	20 (13)	5 (1)	V4–V6	Baseline	After induction remission chemotherapy, the fecal microbiota of the patients changed significantly
Yu et al. (2021)	China	AML	29 (19)	33 (17)	V4	Baseline	The results indicate that the gut microbiota was altered in ML patients compared to that of healthy subjects
Liu et al. (2021)	China	ALL	58 (34)	NA	V1–V9	Baseline	Gut microbiota alteration was associated with chemotherapy-induced pneumonia in pediatric ALL patients
Rashidi et al. (2022)	America	AML	52 (NA)	NA	V4	Baseline, During chemotherapy	The intestinal microbiota of AML patients is destroyed during induction chemotherapy, and this destruction is persistent and cannot return to normal after the end of chemotherapy

When gut microbiota dysbiosis causes the destruction of intestinal epithelial barrier, some gut microbiota enters the blood or local lymph nodes, leading to an inflammatory immune response through metabolic disorders, activation of immune cells and changes in key intracellular signaling pathways, which may contribute to the development of cancer (Ivanov et al., 2008; Gaboriau-Routhiau et al., 2009; Tsilimigras et al., 2017). A study found that bacterial composition was significantly different between an xenotransplant pediatric ALL mouse model and control group in feces and small intestine, such as the increased proportion of bacteria with conversion function of dietary flavonoids in a mouse model of ALL (Song and Gyarmati, 2020). Dietary bioflavonoids were demonstrated to induce mixed lineage leukemia (MLL) gene cleavage by targeting topoisomerase II, and they may lead to childhood leukemia (Strick et al., 2000), indicating that the abnormal composition of gut microbiota is associated with the pathogenesis of leukemia. Changes in the gut microbiota may also be associated with genetic susceptibility to ALL, in mouse models of ALL, and differences in its composition were detected between healthy mice and mice genetically predisposed to leukemia (Vicente-Dueñas et al., 2020).

## Relationship between gut microbiota and the treatment of acute leukemia

### Chemotherapy

**Figure 1:**
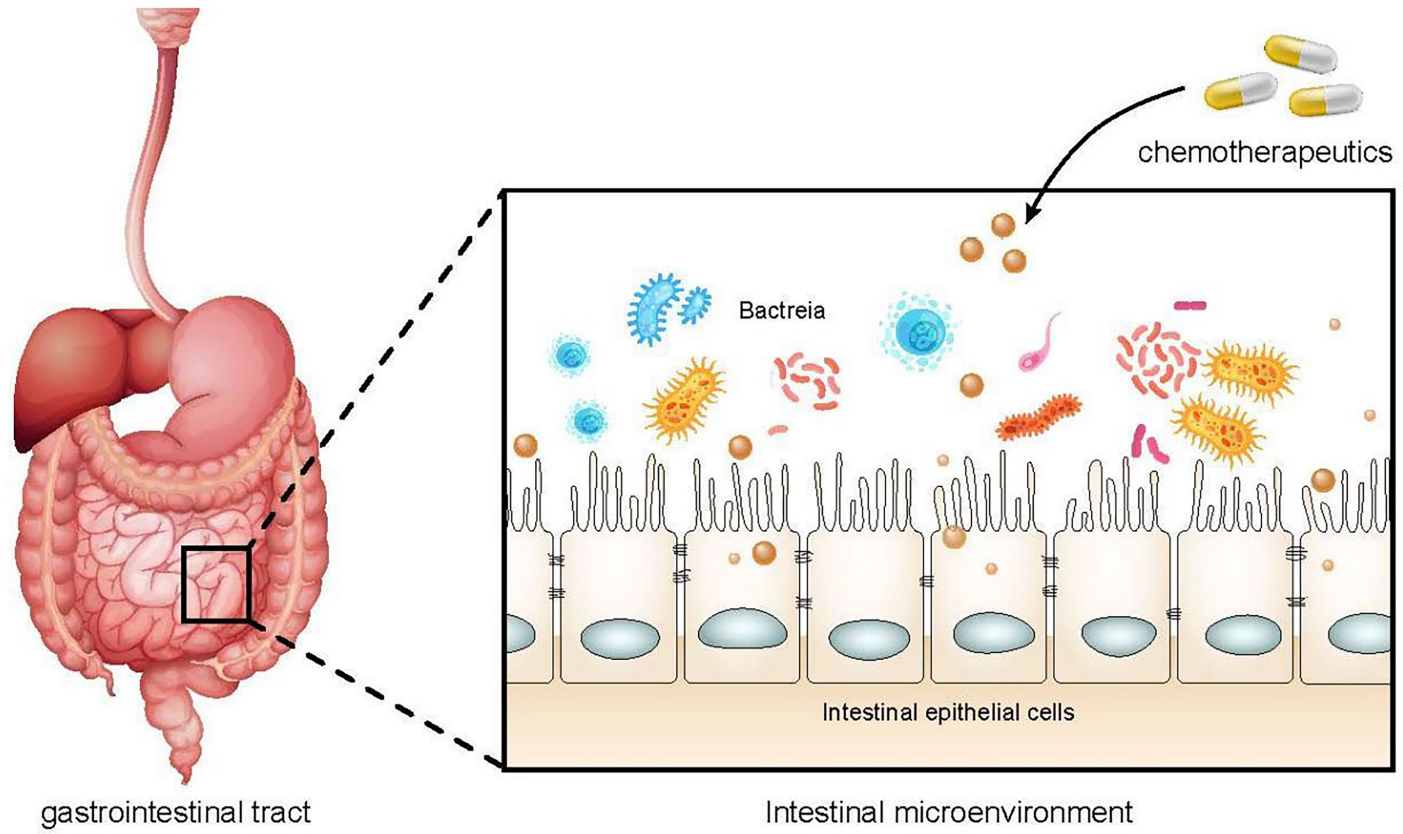
The effect of chemotherapy drugs on gut microbiota. The use of chemoherapeutic drugs can damage intestinal epithelial cells, alter intestinal permeability, and reduce the protective function of the intestinal barrier, resulting in intestial dysbiosis and an increased risk of infection.

The correlations of chemotherapeutic drugs and gut microbiota are mutual. Chemotherapy drugs can directly act on the gut microbiota or damage the intestinal epithelial cells and destroy the intestinal barrier, resulting in the imbalance of the microbiota (Figure 1). Similarly, dysregulated gut microbiota may affect the absorption and metabolism of chemotherapeutic drugs, increasing their toxicity and reducing their efficacy.

A growing body of evidence demonstrated that gut microbiota is closely associated with drug efficacy and side effects, and the interaction between gut microbes and commonly used non-antibiotic drugs is mutual: the composition of the gut microbiome can be affected by drugs, and vice versa. The gut microbiome can also influence an individual’s response to a drug by enzymatically altering its structure and altering its bioavailability, bioactivity, or toxicity, which is called pharmacomicrobiology (Lucafo et al., 2020). However, gut microbiota can also directly influence an individual’s response to chemotherapeutic drugs in the treatment of AL. Methotrexate (MTX) is one of the most clinically crucial and potent anticancer drugs, which is widely used for the treatment of leukemia due to blocking folate metabolism. MTX can induce distinct intestinal impairment, including the atrophy of jejunal villi, increasing goblet cells, and collapse of muscularis mucosa. Moreover, the MTX-induced intestinal mucosal injury promotes a series of inflammatory responses, including the increase of inflammatory factors, and dysregulation of macrophages (increase of M1 phenotype and decrease of M2 phenotype) in lymph nodes and spleen. Besides, MTX can gradually induce an alteration in population, diversity, and composition of the gut microbiota in mice, especially the distinct decrease of *Bacteroidales. Bacteroides fragilis* also significantly decreases, whereas the *Bacteroides fragilis* supplementation can ameliorate the inflammatory response and polarization imbalance of macrophages induced by MTX (Zhou et al., 2018). Changes in the gut microbiota were also observed in some *in vivo* experiments in mice models (Huang et al., 2020; Letertre et al., 2020).

Cyclophosphamide (CTX) is another broad-spectrum antitumor drug and has a curative effect on leukemia and solid tumors. One important study demonstrated that the intestinal epithelial barrier was impaired after treatment with non-high-dose CTX in mice, which was manifested by discontinuity of epithelial barrier, shortening of villi, interstitial edema, focal accumulation of monocytes in lamina propria, and increase of goblet cells and Paneth cells (Viaud et al., 2013). Daillère et al. (2016) identified two bacterial species, *Enterococcus hirae* and *Barnesiella intestinihominis* that are involved in CTX therapy. Whereas *E. hirae* could be translocated from the small intestine to secondary lymphoid organs and increased the intratumoral CD8/Treg ratio. *Barnesiella intestinihominis* was accumulated in the colon and promoted the infiltration of interferon gamma (IFN-γ)-producing gamma delta T (γδT) cells in cancer lesions, and they can improve the efficacy of the most common alkylated immunomodulatory compounds. The above mentioned pathological changes increase the intestinal permeability, facilitate intestinal bacterial translocation, and eventually lead to bloodstream infection (BSI). Viaud et al. (2013) also found a significant change of the microbiota composition in the small intestine and a significant translocation of commensal bacteria in most mice treated with CTX, and some gram-positive bacteria species (e.g., *Lactobacillus johnsonii*, *Lactobacillus murinus* and *Enterococcus hirae*) were detected in lymphoid organs such as mesenteric lymph nodes and spleens. Furthermore, a previous study indicated that gut microbiota was correlated with the antitumor effect induced by chemotherapeutic agents. CTX induced the conversion of CD4^+^ T cells into type-1 T helper (Th1) and Th17 cells, which is closely associated with gram-positive bacterial species present in the small intestine and lymphatic organs. Antibiotic treatment can decrease the CTX-mediated inhibition of tumor growth, whereas adoptive transfer of pathogenic Th17 (pTh17) cells can eliminate this negative effect of antibiotics, suggesting that gut microbiota may contribute to CTX-mediated anticancer immune responses through pTh17 cells, and gram-positive bacteria may contribute to the formation of anticancer immune responses by stimulating Th17 cells (Viaud et al., 2013). A recent large-scale study (Zimmermann et al., 2019) involving 76 diverse human gut bacteria and 271 oral drugs indicated that the metabolic effects of specific gut bacteria on drugs have a significant influence on chemotherapy. In this study, dexamethasone was metabolized by *Clostridium scindens in vitro*, and it was metabolized (side-chain cleaving) by an intestinal microbe *in vivo*, which may influence the serum metabolism level. Other corticosteroids, such as prednisone, may be metabolized by *Clostridium scindens.* Their findings suggested that the metabolism of several drugs is chemically modified by microbiota. High-throughput gene sequencing was combined with mass spectrometry to systematically identify microbial gene products that metabolize drugs, and drug metabolic activities of human gut bacteria and microbiota can be predicted based on their genomic contents. When the interpersonal variation of microbiota is correlated with differences in drug metabolism, drug responses may be safer and more efficient through adjustment of exogenous microorganisms.

### Immunotherapy

Although chemotherapy and hematopoietic stem cell transplantation for AL have achieved a remarkable therapeutic efficacy, some patients still encounter with troubles, such as primary or secondary drug-resistance, relapse after remission, and serious complications. In recent years, novel cancer immunotherapeutic methods with a high specificity and a low toxicity have shown a great potential for the treatment of refractory or relapsed AL. At present, the main immunotherapy for AL contains chimeric type of antigen receptor (CAR) T cell therapy, antibody-drug conjugate (ADC), bispecific T cell engager (BiTE), immune checkpoint inhibitors (ICIs), natural killer (NK) cell therapy, dendritic cell (DC) therapy, etc. A growing body of evidence showed that microbiota may alter the response to cancer immunotherapy. ICIs prevent the binding of inhibitory molecules (e.g., CTLA4, PD-1/PD-L1, LAG-3, and TIM-3) to their ligand receptors to activate the suppressed immune system and promote the removal of malignant cells (Seidel et al., 2018). A remarkable study demonstrated that the composition of gut microbiota (distinct *Bacteroides* species) affected the anti-tumor efficacy of anti-CTLA4 in mice with tumor. It was found that in mice and patients, T cell responses specific for *B. thetaiotaomicron* or *B. fragilis* were associated with the efficacy of CTLA-4 blockade. Tumors in antibiotic-treated or germ-free mice did not respond to CTLA blockade. This defect was overcome by gavage with *B. fragilis*, by immunization with *B. fragilis* polysaccharides, or by adoptive transfer of *B. fragilis–*specific T cells. Fecal microbes from individuals to mice transplanted confirmed that treatment of melanoma patients with anti-CTLA-4 antibody is beneficial to the growth of *B. fragilis* and has anticancer properties (Vétizou et al., 2015). Another study reported that special taxa can promote the anti-tumor efficacy of anti-PD-L1 in mice with melanoma (Sivan et al., 2015). Studies confirmed that a higher diversity of gut microbiota and a higher abundance of particular taxa were associated with positive responses to ICIs in patients with different types of cancer, such as melanoma, non-small cell lung carcinoma, renal cell carcinoma, and urothelial carcinoma (Dubin et al., 2016; Frankel et al., 2017; Routy et al., 2018; Jin et al., 2019). Kuczma et al. found that the loss of gut microbiota caused by administration of antibiotics reduced the therapeutic efficacy of adoptive T cell therapy in mice with colorectal tumors, while did not affect the efficacy of CD19-targeted CAR-T cell therapy in mice with B-cell lymphoma (Kuczma et al., 2017). Abid et al. speculated that gut microbiota may potentially enhance CAR-T response without clear evidence (Abid et al., 2019). Additional relevant studies are required to confirm the above-mentioned assumption. Establishment of a mouse model of liver cancer revealed that the altered gut microbiota induced by vancomycin might have anti-cancer effects by increasing the number of hepatic NKT cells and reducing the level of secondary bile acid metabolism (Ma et al., 2018). To explore the role of the gut microbiota in response to CD19-targeted CAR-T cell therapy, Smith et al. (2022) first conducted a multicenter retrospective study, and it was found that the antibiotic exposure of patients receiving CAR-T cell therapy was associated with worse overall survival (OS). Then, they prospectively collected stool samples from 48 patients with leukemia or lymphoma, and demonstrated that patients receiving CAR-T cell therapy with an abnormally low alpha diversity and a higher abundance of *Ruminococcus, Bacteroides* and *Faecalibacterium* were associated with the higher efficacy of CD19-targeted CAR-T cell therapy. Moreover, additional microbiota data at different treatment time points (e.g., after CAR-T cell therapy and during follow-up) may assist clinicians to better define the role of gut microbiota in CAR-T cell therapy. Further studies should also investigate specific mechanisms of fecal microbiota regulation in preclinical models, such as animal and cell experiments, to explore the interaction of bacterial taxa and bacterial metabolites in the immune system, in order to improve patient outcomes after immunotherapy.

## Effects of gut microbiota on the prognosis of acute leukemia

To our knowledge, chemotherapy is one of the treatment options for AL worldwide, and all pediatric and adult cases with AL receive chemotherapy. However, intensive chemotherapy may have a long-term effect on the gastrointestinal microbiota through a direct damage to the intestinal tract and an indirect influence of the immune system, and the therapeutic or preventive antibiotics used during chemotherapy may also affect the gut microbiota. Thus, the gut microbiota of AL patients may significantly vary after chemotherapy, which has been confirmed by a number of studies. Hakim et al. (2018) found that the diversity of gut microbiota significantly decreased and the abundance of some bacteria, such as *Bacteroidetes*, reduced, whereas that of other bacteria, such as *Clostridiaceae* and *Streptococcaceae*, increased in 199 children with ALL who underwent intensive induction. Multiple studies have also shown that the diversity of gut microbiota decreased during induction chemotherapy in children with ALL, especially *Bacteroides* genus, and the composition of gut microbiota was recovered to the same as that of healthy group after cessation of thermotherapy, while partly accompanied by different levels of abundance (Chua et al., 2020; De Pietri et al., 2020). In addition, similar studies on patients with acute myelocytic leukemia (AML) have yielded consistent results. These studies have found a significant decrease in alpha diversity and beta diversity of gut microbiota in patients with AML after chemotherapy, which is associated with the use of antibiotics, particularly carbapenems (Galloway-Peña et al., 2016; Galloway-Pena et al., 2017; Galloway-Peña et al., 2020). Furthermore, a growing body of evidence demonstrated that the altered gut microbiota is associated with complications after chemotherapy, affecting the prognosis of leukemia patients. The main complications caused by chemotherapy include organ toxicity, neutropenia, infections, gastrointestinal dysfunction, etc. Among them, BSI due to chemotherapy-induced myelosuppression is a common complication and an important cause of high mortality of AL. Song et al. found an increased proportion of opportunistic pathogens (including *Staphylococcus*, *Streptococcus*, *Ralstonia,* and *Lactococcus*) in the feces of ALL mice compared with control mice (Song and Gyarmati, 2020). Once antitumor treatment is initiated, owing to the damage to intestinal epithelial barrier, these pathogens are susceptible to translocation to cause bacteremia and even resistant bacterial infections. Numerous AL-based cohort studies have found that baseline microbiota diversity and composition characteristics prior to chemotherapy are independent predictors of infections during induction chemotherapy. For instance, two studies (Galloway-Peña et al., 2016, 2020) demonstrated that baseline alpha diversity can predict infections during chemotherapy for AML patients. Febrile neutropenia is a common complication in patients with hematologic malignancies receiving chemotherapy, and is associated with high morbidity and mortality (Hakim et al., 2018). A study revealed that the abundance of *Proteobacteria* prior to treatment can predict febrile neutropenia. Besides, changes in gut microbiota are associated with the severity of systemic inflammation and intestinal epithelial loss during chemotherapy (De Pietri et al., 2020). The domination of *Enterococcaceae* or *Streptococcaceae* predicted a higher risk of subsequent infection in ALL patients (Hakim et al., 2018), and a decrease in alpha and beta diversities was corrected with an increased risk of infection within 6 months after therapy (Nearing et al., 2019). Increased temporal variability of gut microbiota was associated with a higher risk of infection at 90 days after induction in AML patients (Galloway-Pena et al., 2017). Therefore, assessing the risk of subsequent infection based on an individual’s microbiota characteristics and timely effective prevention or treatment may reduce mortality in AL patients.

Allogeneic hematopoietic stem cell transplantation (allo-HSCT) is a crucial treatment for AL, especially for patients with relapsed or refractory ALL. It has been found that there are associations between gut microbiota and life-threatening major complications of HSCT, such as graft-versus-host disease (GVHD), infections, and relapse. During allo-HSCT, gastrointestinal mucosa was damaged and intestinal colonizing bacteria were destroyed, resulting in the decrease of microbial diversity. In a cohort study of 80 patients undergoing HSCT, a lower diversity of gut microbiota was related to a higher mortality, suggesting that the microbial diversity was a strong independent predictor of mortality (Taur et al., 2014). Another similar study found that the increased bacterial diversity and genus *Blautia* were correlated with the reduced mortality (Jenq et al., 2015). Several studies have shown that the phylogenetic and functional alterations in gut microbiota were associated with the onset or severity of acute GVHD after allo-HSCT, such as the reduced diversity, some specific bacteria (*Lachnospiraceae*, *Blautia* genus, *Ruminococcaceae*, and anti-inflammatory *Clostridia*), and metabolic changes (the reduced levels of short-chain fatty acid: acetate, propionate, butyrate), which can serve as diagnostic predictors (Doki et al., 2017; Golob et al., 2017; Liu et al., 2017; Mancini et al., 2017; Simms-Waldrip et al., 2017; Payen et al., 2020; Zhou et al., 2020). An important multivariate analysis indicated that a high abundance of *Lactobacillaceae* and a high concentration of human beta-defensin 2 (HBD2) before HSCT were associated with moderate or severe acute GVHD and a high mortality post-HSCT; a high abundance of obligate anaerobes and a rapid recovery of B and NK cells were associated with no or mild acute GVHD and a low mortality post-HSCT; a high abundance of facultative anaerobic bacteria was associated with chronic inflammation (Ingham et al., 2019). Taur et al. pointed out that the reduced diversity of gut microbiota and dominance of opportunistic pathogens, including *Enterococcus, Streptococcus*, and *Proteobacteria,* significantly increased the risk of bacteremia during allo-HSCT (Taur et al., 2012). In a large cohort study (541 patients), Peled et al. reported that the presence or a higher abundance of a bacterial group mainly consisting of *Eubacterium limosum* has a significant relationship with a lower risk of relapse in patients undergoing allo-HSCT (Peled et al., 2017). Therefore, the diversity or composition of gut microbiota or specific species may be regarded as potential biomarkers to predict and prevent complications and prolong OS after allo-HSCT.

## Implications of microbiota-based treatment for acute leukemia

In summary, the microbiota is altered in the onset and treatment process of leukemia, and the alterations may affect the anti-leukemia treatment and prognosis, so as to improve the prognosis of patients by restoring the altered microbiota. There are several strategies to adjust the gut microbiota, so that the altered gut microbiota may returns to a healthy state or tend to change in favor of the patient.

### Probiotics

Probiotics are active microorganisms that confer health benefits on host by decreasing intestinal dysbiosis, promoting nutrient absorption, protecting intestinal mucosal barrier, and modulating immunity or inhibiting intestinal inflammation. Probiotics have been widely used to manage intestinal side effects caused by antibiotics or other factors. A study demonstrated that a probiotic (*Lactobacillus*) can regulate the diversity and abundance of gut microbiota in mice that received 5-fluorouracil (5-FU; Yeung et al., 2020). In a randomized controlled trial involving 60 children with AL who received chemotherapy, the group of patients who took oral probiotics (*Lactobacillus rhamnosus*) daily showed a significant reduction in most gastrointestinal side effects, including vomiting, nausea, abdominal distension, flatulence, constipation, and abdominal pain (Reyna-Figueroa et al., 2019). In a systematic review, the analysis of 11 studies including 1,557 patients has shown that probiotics reduce both frequency and severity of diarrhea in cancer patients (Redman et al., 2014). However, a meta-analysis of 1,091 patients showed that prophylactic probiotics were not effective in reducing diarrhea induced by any cancer therapy (Wardill et al., 2018). Therefore, there is no consistent conclusion about the side effects of probiotics in cancer patients, and further research needs to be conducted to draw definitive conclusions.

Limited supplementation of probiotics may not be enough to restore gut microbiota dysbiosis secondary to AL, while according to the patient’s specific chemotherapy regimen and changes in the gut microbiota, the selection of individualized probiotics may be beneficial to patients, and the prognosis of the patient’s disease and treatment-related side effects may be improved.

### Fecal microbiota transplantation

Fecal microbiota transplantation (FMT) is an infusion of fecal suspension containing healthy donor microbiota into the gastrointestinal tract of the recipient. FMT has been given as self-administered or medically administered enemas, oral capsules, *via* nasogastric tube, or instilled into the duodenum by upper endoscopy or the right colon by colonoscopy or cecostomy. It can directly change the intestinal microbiota of the recipient and restore its normal composition, thus obtaining therapeutic effects. FMT has been highly regarded since it was approved by the US Food and Drug Administration in 2013 for the treatment of recurrent and refractory Clostridium difficile infections, and since then, its application has expanded rapidly and widely, not only for gastrointestinal related diseases, but also for parenteral diseases. For different hosts and diseases, FMT can be personalized to different patients and conditions (Vindigni and Surawicz, 2017; Wang J. W. et al., 2019; Tkach et al., 2022).

Studies have shown that the post-antibiotic intestinal microbiota of the human and murine can recover more quickly with autologous FMT than with multi-strain probiotics (Suez et al., 2018). Therefore, FMT is a faster and more effective method to restore the altered gut microbiota, and many clinical trials have confirmed the therapeutic effect of FMT. In a clinical trial including four acute leukemia patients undergoing allo-HSCT, who suffered from refractory diarrhea because of refractory intestinal infection or intestinal GVHD, FMT was a successful and safe treatment (Wang Q. et al., 2019). In addition, FMT is recommended for the treatment of multiple recurrent *Clostridioides difficile* infection (CDI) in adult and children who have failed appropriate antimicrobial therapy (McDonald et al., 2018). A case report presented a successful FMT treatment in a 27 year-old AML patient with prolonged and severe neutropenia. The patient developed fulminant CDI refractory to maximal antibiotic treatment, then appeared high fever, diffuse abdominal pain, watery diarrhea, colitis and other serious clinical symptoms, whereas his clinical symptoms improved after FMT therapy without further recurrence.(Lee et al., 2020) Two other similar case studies also reported that FMT was successful in eliminating multidrug-resistant pathogens and treating recurrent CDI after allo-HSCT in ALL patients (de Castro et al., 2015; Innes et al., 2017). In addition, FMT intervention achieved good results in restoring microbial diversity and reconstructing gut microbiota composition after HSCT (DeFilipp et al., 2018; Taur et al., 2018), as well as in treating four patients with steroid-resistant gut aGVHD (Kakihana et al., 2016). In order to evaluate the efficacy of autologous fecal microbiota transfer (AFMT) in dysbiosis correction and multidrug-resistant bacteria eradication, 25 AMl patients treated with (de Vos et al., 2022) intensive chemotherapy and antibiotics were recruited in a multicenter study, they showed that AFMT appears to be safe and effective for gut microbiota restoration in patients receiving intensive induction chemotherapy and antibiotic for AML (Malard et al., 2021).

### Diet regulation and prebiotics

In 2017, the International Scientific Association for Probiotics and Prebiotics (ISAPP) consensus panel defined a prebiotic as follows: a substrate that is selectively utilized by host microorganisms conferring a health benefit (Gibson et al., 2017). Some substances present in the diet, such as soybean extracts, koji glycosylceramides, grape extracts, tea polyphenols, and seaweed extracts, can be considered as potential prebiotics, because they can selectively stimulate the proliferation of beneficial bacteria in the intestine (Cheng et al., 2022). The effects of prebiotics are indirect, and their positive effects are exerted by affecting the composition of gut microbiota.

A recent study suggested that the use of appropriate prebiotics during chemotherapy may reduce the therapeutic side effects. Establishment of a mouse model revealed that short-term dietary restriction significantly alleviated the intestinal fatal damage in mice after high-dose MTX exposure by increasing the *Lactobacillus genus* to reduce intestinal inflammation and protect PCNA-positive cells in basal crypt, as well as the function of intestinal stem cells (Tang et al., 2020). Importantly, *Lactobacillus rhamnosus* supplementation plays the same role as dietary restriction, suggesting that dietary regulation is a viable method to restore the altered microbiota after treatment. Taskin et al. (2017) found in a study on mice that lactulose significantly improved MTX-induced liver damage. Similarly, a recent study conducted by Zhou et al. (2021) pointed out that polysaccharides and ginsenosides extracted from American ginseng had a certain protective effect on CTX therapy. They restored the composition of gut microbiota and increased various beneficial mucosa-associated bacterial taxa (*Clostridiales*, *Bifidobacterium*, and *Lachnospiraceae*), while decreased harmful bacteria (*Escherichia-Shigella* and *Peptococcaceae*). Besides, specific nutrients may play an important role in adjusting gut microbiota. For instance, a study found that melatonin supplementation increased the diversity of gut microbiota and regulated the composition, such as enrichment of the abundance of *Lactobacillus* in mice (Ren et al., 2018), indicating that supplementing food enriched in melatonin is conducive to the early reconstruction of gut microbiota after chemotherapy. Another study confirmed that soy-whey blended protein can significantly increase the diversity of gut microbiota and effectively improve muscle status in AL survivors (Ren et al., 2020). In addition, enteral nutrition was given to promptly restore the gut microbiota post-HSCT (D'Amico et al., 2019). Therefore, a proper diet and supplementation of prebiotics are also an alternative treatment for the altered gut microbiota.

## Conclusion

Recent studies have explored the role of gut microbiota in AL. Gut microbiota contributes to the risk of leukemia by interacting with the immune response. Gut microbiota may affect the therapeutic efficacy by influencing the metabolism of chemotherapeutic drugs and improving the patient’s response to immunotherapy. In turn, these anti-cancer treatments affect the diversity and composition of gut microbiota. The diversity and abundance of gut microbiota or specific taxa can be used as strong independent predictors for infection, recurrence, GVHD, and death in AL patients receiving therapy. It is potential to treat AL by regulating gut microbiota. Microbiota replacement therapies and supplementing microbiota with specific probiotics and dietary regulation have become effective measures to prevent or treat leukemia-associated complications and to improve prognosis of patients with AL. However, it remains elusive which specific taxa may play a key role in initiation, progression, and treatment of AL. The exploration of these special microbiota needs further study in the future.

## Author contributions

CZ drafted the manuscript. YZ revised the manuscript and prepared illustrations. AZ conceived the topic and critically revised the manuscript. All authors contributed to the article and approved the submitted version.

## Funding

This study was financially supported by Key Research and Development Program of Shandong Province (grant no. 2019GSF108060) and Shandong Provincial Natural Science Foundation (grant no. ZR202010220039).

## Conflict of interest

The authors declare that the research was conducted in the absence of any commercial or financial relationships that could be construed as a potential conflict of interest.

## Publisher’s note

All claims expressed in this article are solely those of the authors and do not necessarily represent those of their affiliated organizations, or those of the publisher, the editors and the reviewers. Any product that may be evaluated in this article, or claim that may be made by its manufacturer, is not guaranteed or endorsed by the publisher.

## Supplementary material

The Supplementary material for this article can be found online at: https://www.frontiersin.org/articles/10.3389/fmicb.2022.1045497/full#supplementary-material
