# Supplementary figures and images for: Gut microbiota in acute leukemia: Current evidence and future directions
Source: Front Microbiol. 2022 Dec 1;13:1045497. doi: 10.3389/fmicb.2022.1045497 (PMC9751036; doi:10.3389/fmicb.2022.1045497)

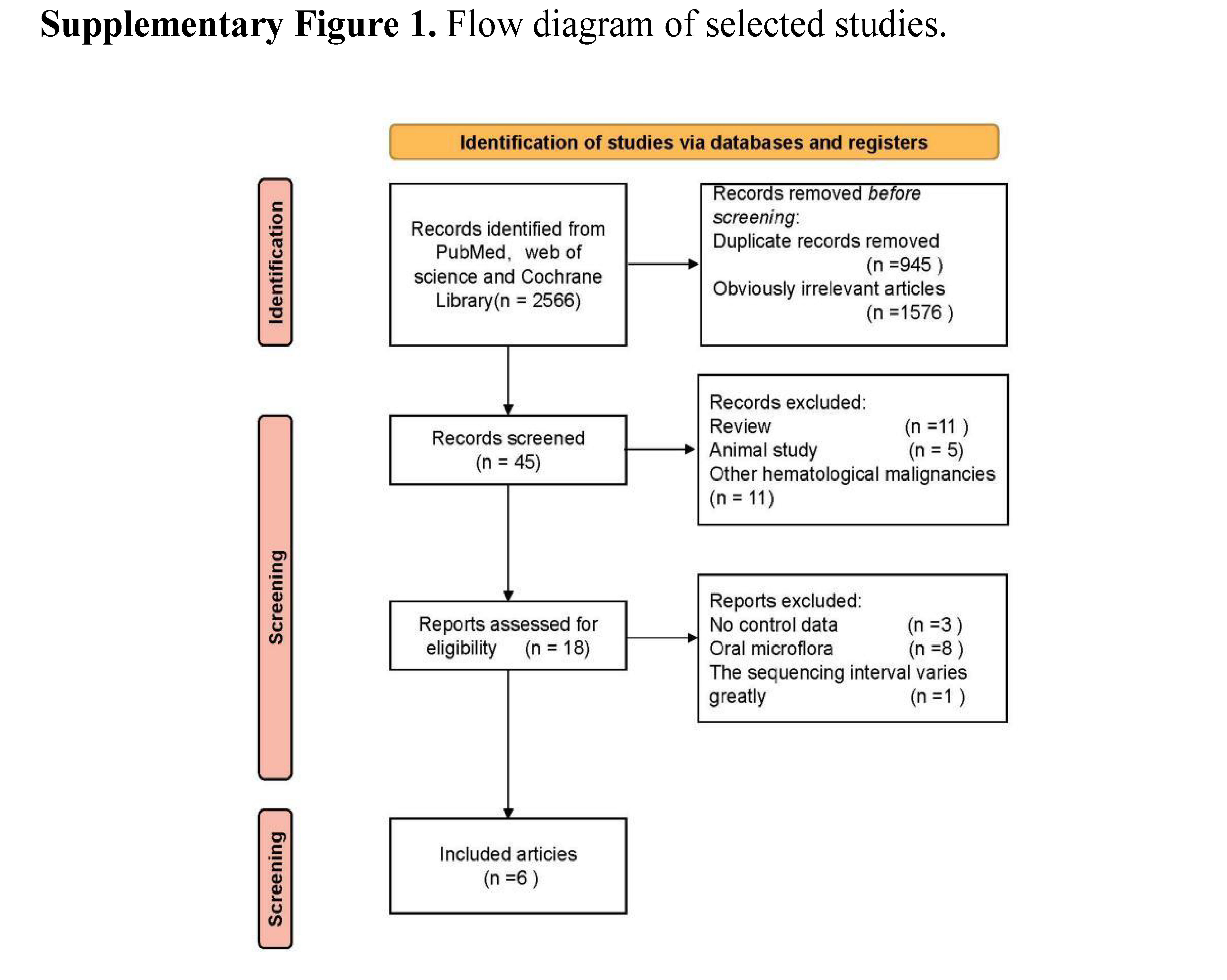

Supplement: Supplementary file 1 [file Image_1.TIF]

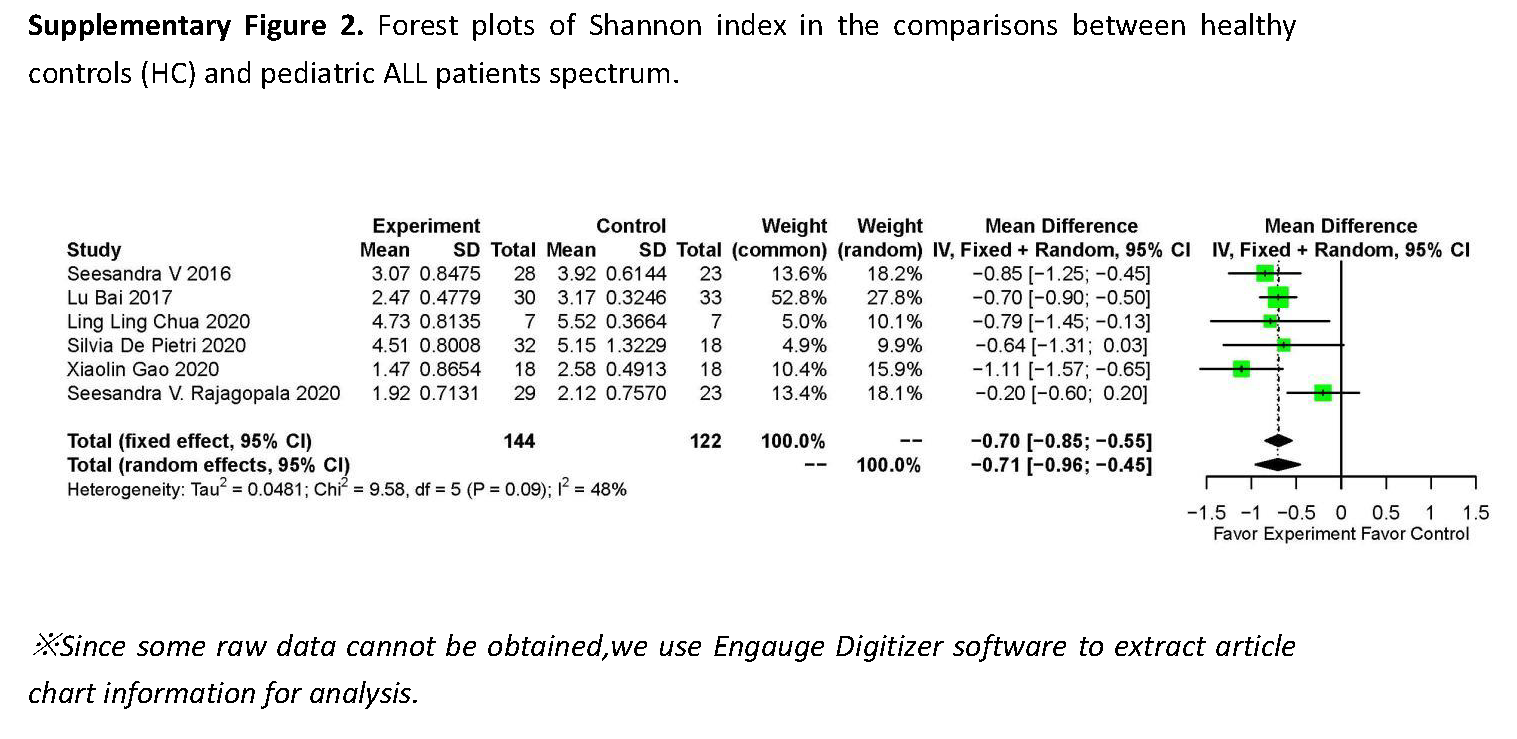

Supplement: Supplementary file 2 [file Image_2.TIF]
